# Comparing ChatGPT’s ability to write and review papers: then what?

**DOI:** 10.3325/cmj.2024.65.397

**Published:** 2024-08

**Authors:** Shigeki Matsubara

**Affiliations:** Department of Obstetrics and Gynecology, Jichi Medical University, Tochigi, Japan; *matsushi@jichi.ac.jp*

To the Editor,

Kadi and Aslaner (1) presented interesting data. In writing, ChatGPT could generate a case report, which, however, failed to reach the level considered acceptable by humans. In reviewing, ChatGPT could not detect mistakes intentionally made by humans. The authors stated (in the first paragraph of the Discussion) that ChatGPT performed better in writing than in reviewing. I have some concerns.

First, in this experiment, writing papers heavily relied on ChatGPT, which is impractical, because few, if any, individuals use ChatGPT in such a manner. I have consistently argued against using ChatGPT for paper writing (2-4). However, if one must use ChatGPT to write a case report, it is essential to provide specific input, according to the case-report writing fundamentals (5). Inputs should typically include the following five: 1) a synopsis, 2) known information regarding the theme, 3) the problem to be clarified, 4) a summary of the patient course, and 5) points to be discussed (Table 1). At the paper writing stage, whether done by humans independently or with the assistance of ChatGPT, one usually has already outlined points 1-5. Let us input these points and then evaluate the ChatGPT-generated case report. Similarly, in reviewing, if one changed “title,” input “title OK?”. Then, one could evaluate ChatGPT’s ability more precisely. I respectfully claim: the present experiment simply showed that inadequate inputs result in poor outputs.

**Table 1:** An example of what to input when tasking ChatGPT with writing a case report

1. Synopsis: Procedure X* is beneficial for condition Y*.
2. Known: Procedure X is commonly used for condition Z*.
3. Problem: How the current case illustrates the applicability of procedure X to condition Y.
4. Summary of the patient's course.
5. Discussion points:
i) The challenges associated with condition Y.
ii) Resemblance of pathophysiology between Y and Z.
iii) How procedure X improved the condition for this particular patient (with Y).
iv) Outlining the merits over demerits of procedure X.
v) Adding ethical considerations.

*For example, one can assume X, Y, and Z as follows. X = pelvic artery embolization; Y = significant ovarian bleeding in a woman with a frozen pelvis (difficult surgery expected); Z = marked traumatic pelvic bleeding.

Why could one conclude that ChatGPT is superior at writing compared with reviewing? Input necessary and adequate information, and then evaluate the outputs in an item-by-item manner (ie, grammar, context, consistency, references, and other factors), which will make a more conclusive determination possible. The present study design does not definitively answer the question of whether ChatGPT performs better at writing or reviewing tasks.

Third, and more importantly, I question the authors’ true intentions. They stated, “With the current technological infrastructure, ChatGPT is not capable of producing logical text.” Essentially, their study implies that, given specific inputs, ChatGPT produces corresponding outputs. The fundamental question lies in their stance: when ChatGPT advances to the point of generating a “complete” case report or review with adequate inputs, will they fully utilize it or use it solely as an advisory tool? This distinction is crucial. I believe that writing and reviewing are exclusive human domains (2-4), and therefore, I align with the latter. How do Kadi and Aslaner consider this?
